# New Insight of Circular RNAs' Roles in Central Nervous System Post-Traumatic Injury

**DOI:** 10.3389/fnins.2021.644239

**Published:** 2021-03-23

**Authors:** Lvwan Xu, Xin Ye, Jinjie Zhong, Ying-ying Chen, Lin-lin Wang

**Affiliations:** ^1^Department of Basic Medicine Sciences, and Department of Orthopaedics of Sir Run Run Shaw Hospital, Zhejiang University School of Medicine, Hangzhou, China; ^2^Department of Neurosurgery, Sir Run Run Shaw Hospital, Zhejiang University School of Medicine, Hangzhou, China; ^3^Department of Basic Medicine Sciences, and Department of Obstetrics of the Second Affiliated Hospital, Zhejiang University School of Medicine, Hangzhou, China

**Keywords:** central nervous system, traumatic brain injury, spinal cord injury, circular RNAs, inflammation, apoptosis

## Abstract

The central nervous system (CNS) post-traumatic injury can cause severe nerve damage with devastating consequences. However, its pathophysiological mechanisms remain vague. There is still an urgent need for more effective treatments. Circular RNAs (circRNAs) are non-coding RNAs that can form covalently closed RNA circles. Through second-generation sequencing technology, microarray analysis, bioinformatics, and other technologies, recent studies have shown that a number of circRNAs are differentially expressed after traumatic brain injury (TBI) or spinal cord injury (SCI). These circRNAs play important roles in the proliferation, inflammation, and apoptosis in CNS post-traumatic injury. In this review, we summarize the expression and functions of circRNAs in CNS in recent studies, as well as the circRNA–miRNA–mRNA interaction networks. The potential clinical value of circRNAs as a therapeutic target is also discussed.

## Introduction

Central nervous system (CNS) post-traumatic injury often leads to persistent neurological damage and devastating consequences, but there is still a lack of effective methods for its treatment (Leng et al., 2019; Tsujioka and Yamashita, 2019). It includes traumatic brain injury (TBI) and spinal cord injury (SCI) and is one of the world's major disease burdens (Warnock et al., 2020). TBI is complex and severe, resulting in neuroinflammation and neurodegeneration (Kempuraj et al., 2020; Zheng et al., 2020). It is a leading cause of death and disability (Yu et al., 2017; Radabaugh et al., 2020). Similarly, SCI is a destructive and traumatic injury that involves primary and secondary injury (Wang and Song, 2019; Ma et al., 2020). It can lead to loss of sensory, motor, and autonomic nerve function (Chen et al., 2019a; Reis et al., 2020; Wang et al., 2020c). The regeneration capacity of the CNS is very limited (Ma et al., 2019; Radabaugh et al., 2020). Recently, more and more studies have explored the expression patterns and functional characteristics of microRNA (miRNA) and lncRNA in CNS post-traumatic injury. It has been found that they are involved in the process of post-traumatic inflammation, apoptosis, and autophagy of the CNS through epigenetic regulation (Nagalakshmi et al., 2018; Yang et al., 2018a; Ge et al., 2019; Li et al., 2019; Yi et al., 2019; Wang et al., 2020b). However, the endogenous mechanisms that mediate CNS post-traumatic injury are still obscure.

Circular RNAs (circRNAs) are one of the noncoding RNAs. They have no 5′-3′ polarity, 5′ capping, and 3′ polyadenylation. Noncanonical alternative splicing connects the splicing donor to the upstream splicing recipient, forming a covalent closed ring (Li et al., 2016). In mammalian cells, circRNAs are endogenous, abundant, conserved, and stable (Wu and Yang, 2015). Although most functions remain unknown, circRNAs have been shown to function as templates for viroid and viral replication, as intermediates in RNA processing reactions, as regulators of transcription in cis, as snoRNAs, and as miRNA sponges (Lasda and Parker, 2014; Enuka et al., 2016).

In the past few years, due to the advance in second-generation sequencing (Enuka et al., 2016), microarray analysis, and bioinformatics, more and more studies have explored the expression and functions of circRNAs in CNS diseases, such as Alzheimer's disease (Zhang et al., 2019), cerebral ischemic stroke (Han et al., 2018), brain tumor (Yang et al., 2018b), and multiple sclerosis (Cardamone et al., 2017). Therefore, researchers speculate that circRNAs may also play important roles in CNS post-traumatic injury. They designed experiments to explore this. The results showed that circRNAs differentially expressed in both TBI and SCI models, and these studies further explored the functions of circRNAs.

In this review, we discussed the role of circRNAs in TBI and SCI and compared their expression profiles. These circRNAs play important roles in the proliferation, inflammation, apoptosis, and post-traumatic injury. We also discussed the clinical significance of using circRNAs as therapeutic targets for CNS post-traumatic injury.

## Circular Expression Profiling in CNS Post-Traumatic Injury

In most studies, the expression profile of circRNAs was obtained by RNA sequencing or microarray and then verified by reverse transcription-quantitative PCR (RT-qPCR). Different kinds of models are used and the results are shown in Table 1.

**Table 1:** Circular RNA (circRNA) expression profiles in CNS post-traumatic injury.

**Models**			**Samples**	**Methods**	**Filtering criteria**	**Up regulated**	**Down regulated**	**References**
Mice	3 h after TBI	FPI	Exosomes	RNA sequencing	*p* ≤ 0.05	155 circRNAs	76 circRNAs	Zhao et al., 2018
				RT-PCR	FC ≥ 2			
Rats	3 h after TBI	FPI	Hippocampus	microarray	*p* < 0.05	98 circRNAs	94 circRNAs	Xie et al., 2018
					FC ≥ 1.5			
Mice	6 h after TBI	CCI	Parietotemporal	RNA sequencing	*p* < 0.05	98 circRNAs	93 circRNAs	Jiang et al., 2019
			cortex	RT-PCR	FC ≥ 2			
Mice	1 days after	CCI	Brain cortex	RNA sequencing	*p* < 0.05	5 circRNAs	11 circRNAs	Chen et al., 2019b
	TBI				FC > 2			
Rats	2 h after	Contusion	T10 spinal cord	Microarray	*p* < 0.05	1,101 circRNAs	897 circRNAs	Liu et al., 2020
	SCI			RT-PCR	FC ≥ 2			
Rats	6 h after SCI	Contusion	T9 spinal cord	Microarray	*p* ≤ 0.05	99 circRNAs	51 circRNAs	Zhou et al., 2019
				RT-PCR	FC ≥ 2			
Mice	3 days after	Contusion	T9–10 spinal cord	Microarray	*p* < 0.05	909 circRNAs	222 circRNAs	Yao et al., 2020
	SCI			RT-PCR	FC > 2			
Mice	3 days after SCI	Contusion	T8–10 spinal cord	Microarray	*p* < 0.05	/	/	Wang et al., 2020d
Rats	3 days after	Contusion	T10 spinal cord	Microarray	*p* < 0.05	415 circRNAs	1,261 circRNAs	Qin et al., 2018
	SCI			RT-PCR	FC ≥ 2			
Rats	7 days after SCI	Contusion	T9–10 spinal cord	Microarray RT-PCR	*p* < 0.05	/	/	Chen et al., 2020

*TBI, traumatic brain injury; SCI, spinal cord injury; FPI, fluid pressure injury; CCI, controlled cortical impact; FC, fold change*.

### CircRNA Expression Profile in TBI

The fluid pressure injury (FPI) method was adopted to make the TBI model. After drilling a hole in the skull, a metal pendulum was lowered to strike the piston, causing a moderate fluid pressure injury by injecting a small amount of saline into the closed skull cavity. The two studies mentioned below took this approach (Xie et al., 2018; Zhao et al., 2018). Zhao and colleagues extracted mice exosomes from the brain extracellular space 3 h after TBI. Through high-throughput sequencing, they found 231 circRNAs with significantly differentially expressed expressions, of which 155 expressions were upregulated and 76 expressions were downregulated (Zhao et al., 2018). In another study, Xie et al. also explored what happened 3 h after TBI using rat hippocampus. Through circRNA microarray, they found 192 circRNAs with significantly differentially expressed expressions, of which 98 expressions were upregulated and 94 expressions were downregulated (Xie et al., 2018). Bioinformatics analysis including GO terms and KEGG pathway showed that circRNAs extracted from exosomes were involved in “dendrite development,” “cytoplasmic,” “protein binding,” and “glutamatergic synapse,” while in the hippocampus, they participated in the “generation of neurons,” “synapse,” “small GTPase binding,” and “axon guidance.” These results suggest that circRNAs are actively involved in regulating the repair process of neurons, dendrites, and synapses after TBI.

In addition to FPI, the controlled cortical impact (CCI) approach has also been adopted. The electromagnetic device is set in advance for speed, strike depth, and dwell time to complete the CCI. In two experiments using CCI on the cerebral cortex, circRNAs were also differentially expressed after TBI in mice. Six hours after TBI on the left parietotemporal cortex, 98 circRNAs were upregulated and 93 circRNAs were downregulated (Jiang et al., 2019). They were involved in “histone deacetylation,” “histone methyltransferase complex,” “repressing transcription factor binding,” and “natural killer cell-mediated cytotoxicity.” One day after TBI between the bregma and lambda (Rola et al., 2006), 5 circRNAs were upregulated and 11 circRNAs were downregulated (Chen et al., 2019b), participating in “innate immune response,” “extracellular region,” “protein binding,” and “natural killer cell-mediated cytotoxicity.” However, the top 10 upregulated or downregulated circRNAs in these researches about TBI were completely different (Xie et al., 2018; Zhao et al., 2018; Jiang et al., 2019; Chen et al., 2019b). The bioinformatics analysis here suggests that circRNAs might regulate the transcription process and immune system to influence the repair process after TBI.

### CircRNA Expression Profile in SCI

In the SCI model, an impactor is dropped at a certain height onto the spinal cord after a laminectomy. Contusion injury can be caused by regulating different weights and falling heights. The injured spinal cord segments were concentrated in T8–T10 in various studies (Qin et al., 2018; Zhou et al., 2019; Chen et al., 2020; Liu et al., 2020; Wang et al., 2020d; Yao et al., 2020). Two studies explored the expression of circRNAs within a short period after SCI. Liu and colleagues extracted T10 spinal cord of rats 2 h after SCI. Through microarray, they found 1,998 circRNAs with significantly differentially expressed expressions, of which 1,101 were upregulated and 897 were downregulated. According to the enrichment analysis, these differentially expressed circRNAs are involved in “intracellular signal transduction,” “synapse,” “protein binding,” and “mitogen-activated protein kinase (MAPK) signaling” (Liu et al., 2020). MAPK can facilitate neuronal degeneration after SCI and promote cell proliferation (Ye et al., 2020). Zhou et al. extracted T9 spinal cord of rats 6 h after SCI. They found 99 upregulated and 51 downregulated circRNAs. According to the enrichment analysis, the upregulated circRNAs are involved in “gene silencing by RNA,” “centromeric region,” “siRNA binding,” and “peroxisome proliferator-activated receptor (PPAR) signaling pathway.” The PPARγ pathway is related with astrocyte-derived neuroinflammation in CCI rats (Zhong et al., 2019). Activation of PPARs can reduce the proinflammatory cascade (Shafi et al., 2019). Meanwhile, the downregulated circRNAs are involved in the “regulation of synaptic vesicle exocytosis,” “synapse,” “protein kinase binding,” and “extracellular matrix-receptor interaction” (Zhou et al., 2019).

Three studies examined the expression of circRNAs 3 days after SCI, which may indicate that this is a critical time point. The researchers focused on the T8–10 spinal cord segments. Yao et al. found 1,131 circRNAs with significantly differentially expressed expressions, of which 909 expressions were upregulated and 222 expressions were downregulated. These differentially expressed circRNAs are involved in “mitosis,” “dendrite,” “calcium ion binding,” and “glycosphingolipid biosynthesis and extracellular matrix-receptor” (Yao et al., 2020). In another analysis by Wang and colleagues, the differentially expressed circRNAs participated in “protein phosphorylation,” “cytoplasm,” and “protein binding” (Wang et al., 2020d). Meanwhile, Qin et al. found 415 circRNAs were upregulated and 1,261 circRNAs were downregulated. According to the enrichment analysis, the target mRNAs of five candidate circRNAs are involved in “metabolic process,” “intracellular,” “anion binding,” and “adenosine 5′ monophosphate-activated protein kinase (AMPK) signaling pathway” (Qin et al., 2018). AMPK controls the axonal regeneration of sensory neurons in the dorsal root ganglion after SCI and plays an important role in energy metabolism and cellular protein homeostasis (Zhao et al., 2017; Kong et al., 2020). There was also a study of 7 days after SCI. Results showed that differentially expressed circRNAs related with “metabolic process,” “intracellular organelles,” “nucleic acid binding,” and “MAPK signaling pathway” (Chen et al., 2020). These results suggest that circRNAs participate in the transcription, metabolism, and other crucial biological processes in cells to regulate the process of repair after SCI.

The expression of some circRNAs decreased significantly at different time points. CircRNA_009608 decreased significantly at both 2 h and 3 days after SCI (Qin et al., 2018; Liu et al., 2020). The fold change was 11.9 at 2 h and 9.3 at 3 days. The gene symbol of circRNA_009608 is Gnaz, which is capable of coupling neurotransmitter receptors to ion channels in sympathetic neurons (Jeong and Ikeda, 1998). The expression of circRNA_015152 decreased 2 h after SCI and the fold change was 10.2 (Zhou et al., 2019), while the expression of circRNA_015151 also decreased 3 days after SCI and the fold change was 9.5 (Qin et al., 2018). CircRNA_015152 and circRNA_015151 have the same gene symbol Anks1b, which is a major component of the postsynaptic density in excitatory neurons (Dosemeci et al., 2016). The decreased expression of these circRNAs may be a manifestation of continuous inhibition of neurological function, but we need more data to observe their expression trend at different time points.

### Comparison of Similarities and Differences of CircRNAs Between TBI and SCI

SCI and TBI are closely related in the pathological mechanism (Baek et al., 2017). Indeed, studies have shown similarities and differences in the research for circRNAs in these two different kinds of injuries. First, various time points are selected in the experiments. In the modeling of these studies, although they are all processes simulating acute injuries, the time points of sample analysis after TBI injuries are mostly earlier than SCI. Second, the sampling location varies. The samples adopted by TBI include the exosomes, hippocampus, brain cortex, etc., while the SCI samples are mostly concentrated in the spinal segments of T8–10. There are also some common points in the findings of bioinformatics analysis. Some results of TBI and SCI are similar in the existing reports on enrichment analysis of differentially expressed circRNAs. For example, circRNAs in both TBI and SCI participate in “organelle organization” (Zhao et al., 2018; Chen et al., 2020), “synapse” (Xie et al., 2018; Liu et al., 2020), and “protein binding” (Zhao et al., 2018; Liu et al., 2020) in the GO term analysis. They also participated in the “axon guidance” (Xie et al., 2018; Chen et al., 2019b, Chen et al., 2020) in the KEGG pathways.

**Figure 1:**
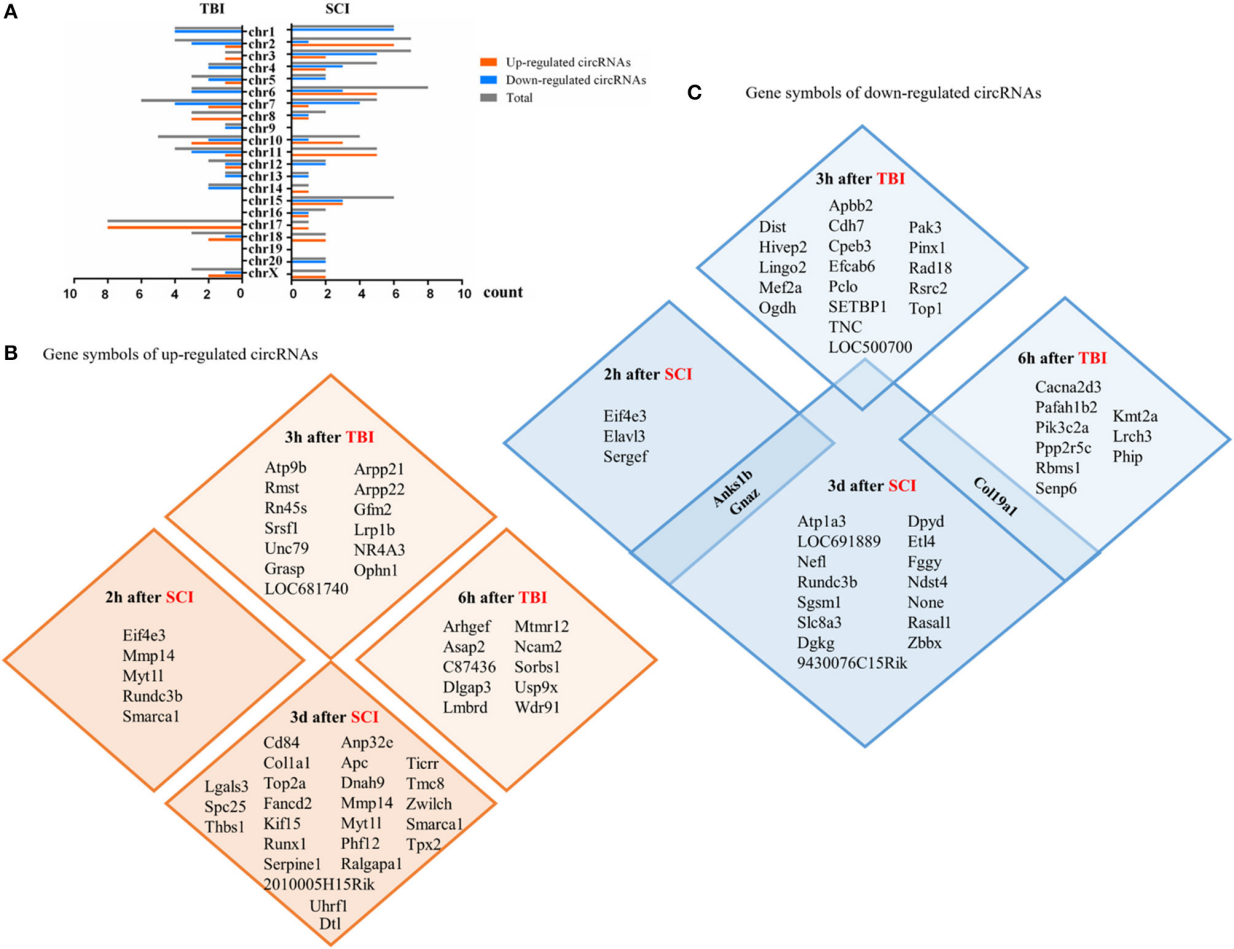
Differentially expressed circular RNAs (circRNAs) (within the top 5–10 upregulated or downregulated in different studies) in CNS post-traumatic injury. **(A)** The distribution of differentially expressed circRNAs on chromosomes. **(B)** The gene symbols of upregulated circRNAs at different time points in the injury site. **(C)** The gene symbols of downregulated circRNAs at different time points in the injury site.

We collected circRNAs that differentially expressed top 10 or top 5. A total of 25 are upregulated and 30 are downregulated in TBI. Thirty-five are upregulated and 35 are downregulated in SCI. The distribution of gene symbols of these circRNAs on chromosomes is shown in Figure 1A. They are distributed across almost every chromosome. In TBI, chromosomes 7, 10, and 11 are mainly concentrated, while in SCI, chromosomes 6, 2, and 3 are mainly concentrated. The gene symbols of upregulated circRNAs are shown in Figure 1B and the gene symbols of downregulated circRNAs are shown in Figure 1C. CircRNA_00180 was significantly decreased 6 h after TBI and its fold change was 5.4 (Jiang et al., 2019). Circ_0008614 was also significantly decreased 3 days after SCI and its fold change was 2.68 (Yao et al., 2020). They share the same gene symbol Col19a1, which is related with encoding nonfibrillar collagen XIX (Su et al., 2010).

## Functional Roles of Specific Circrnas in CNS Post-Traumatic Injury

### Upregulated CircRNAs

The expression of circRNAchr8_87,859,283–87,904,548 increased significantly in the mouse model 6 h after TBI. Its expression rose about fourfold in the damaged areas around the damaged cerebral cortex. The study proved that circRNAchr8_87,859,283–87,904,548 can play a role in increasing the CXCR2 protein by sponging mmu-let-7a-5p. CXCR2 can cause neuropathic pain and inflammatory pain through the interaction between astrocytes and neurons (Cao et al., 2016). Thus, the increase of the circRNAchr8_87,859,283–87,904,548 promotes neuroinflammatory responses and further inhibits the recovery of nervous system function. This suggests that inhibiting the expression of circRNAchr8_87,859,283–87,904,548 may contribute to the recovery after TBI through anti-inflammatory effects (Chen et al., 2019b).

Three days after SCI, the expression of cicRNA_7079 increased significantly in mice. Ying Yao et al. found that cicRNA_7079 could act on the cicRNA_7079–mmu-Mir-6953-5p–Lgals3 axis (Yao et al., 2020). Recent studies have found that Gal-3 can modulate the severity of neuroinflammation after SCI by enhancing the activation of the ROS/TXNIP/NLRP3 signaling pathway (Ren et al., 2019). Meanwhile, in NSC-34 motor neurons, deletion of cicRNA_7079 increases neuronal apoptosis. The results further proves that cicRNA_7079 could play an anti-apoptotic role. This study helps elucidate the molecular mechanisms associated with apoptosis in SCI (Yao et al., 2020).

The study of Jun Chen et al. showed that 7 days after SCI, circRNA_2960 was the top 1 upregulated circRNA. CircRNA_2960 downregulated miRNA_124 and functioned through the interaction network of circRNA_2960–miRNA_124. It was proved that circRNA_2960 could aggravate the inflammatory response and induce apoptosis at the site of injury. The study further demonstrated that blocking the expression of circRNA_2960 could be beneficial to the recovery of spinal cord injured tissues (Chen et al., 2020). However, the functional relationship between miRNA-124 and mRNA remains to be explored by subsequent researches.

**Figure 2:**
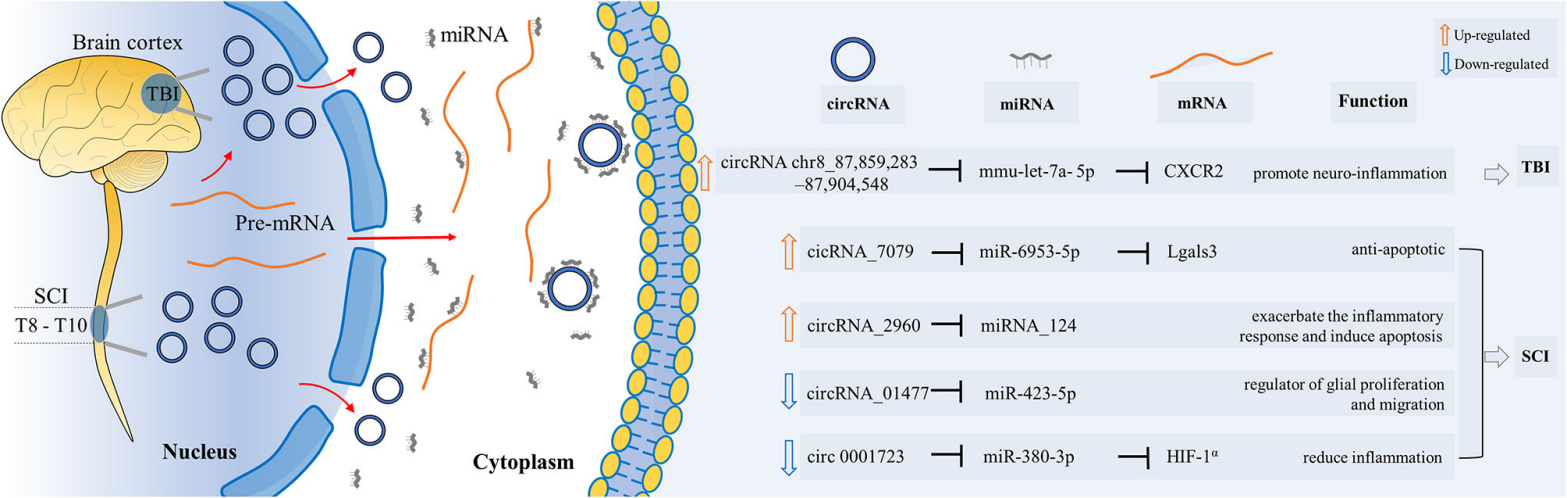
Functional roles of specific circRNAs in CNS post-traumatic injury. CircRNAs play their roles as miRNA sponges through the circRNA–miRNA–mRNA interaction networks. They influenced the biological processes of proliferation, inflammation, apoptosis, etc.

In summary, both circRNAchr8_87,859,283–87,904,548 in the TBI model and circRNA_2960 in the SCI model play roles in promoting inflammation. The three circRNAs mentioned above are upregulated in traumatic CNS injuries (Table 2 and Figure 2). They act on different circRNA–miRNA–mRNA axes to function and are related to the process of inflammation or apoptosis. Other unexplored upregulated circRNAs might play important roles in similar or different biological processes in CNS post-traumatic injury. They may also act in ways other than miRNA sponges, which need to be confirmed by further investigation.

**Table 2:** Functional characterization of the circRNAs in post-traumatic CNS injury.

**Model**	**CircRNA**	**Regulation**	**ceRNA network**	**Functional roles**	**References**
TBI	CircRNA_16895	Down	(Predicted) CircRNA_16895–miRNA–Myo10	Regulate fragment crystallizable gamma receptor (FccR)-mediated phagocytosis pathway	Jiang et al., 2019
TBI	CircRNAchr8_87,859, 283–87,904,548	Up	CircRNAchr8_87,859,283–87,904,548-mmu-let-7a-5p-CXCR2	Promote neuroinflammation	Chen et al., 2019b
SCI	CircRNA_01477	Down	CircRNA_01477–miR-423-5p	Regulator of glial proliferation and migration	Wu et al., 2019
SCI	Circ 0001723	Down	Circ 0001723–miR-380-3p–HIF-1α	Anti-inflammation	Li et al., 2020
SCI	CicRNA_7079	Up	CicRNA_7079–mmu-miR-6953-5p–Lgals3 axis	Anti-apoptotic	Yao et al., 2020
SCI	CircRNA_2960	Up	CircRNA_2960–miRNA_124	Exacerbate the inflammatory response and induce apoptosis at the lesion site	Chen et al., 2020

### Downregulated CircRNAs

CircRNA_16895 was significantly decreased in mice 6 h after TBI, and it was predicted to act on the circRNA_16895–miRNA–Myo10 axis. It might be able to regulate the fragment crystallizable gamma receptor-mediated phagocytosis pathway (Jiang et al., 2019). However, this pathway is still at the predicted stage and has not been confirmed by experiments yet. Future studies are supposed to figure out the function mechanism of circRNA_16895.

The expression of circRNA_01477 decreased significantly in rats after SCI, which serves as a regulator of glial proliferation and migration by acting on the axis of circRNA_01477–miR-423-5p. In this study, Wu et al. (2019) prevented the expression of circRNA_01477 through small interfering RNA. It stunted the proliferation and migration of astrocytes. Subsequently, microarray assay was used to explore the circRNA_01477–miRNA–mRNA network. They found that among the seven validated mRNAs with decreased expression, four were regulated by miRNA-423-5p. Meanwhile, miRNA-423-5p was significantly increased after circRNA_01477 interference. This further indicates that the regulation of the circRNA_01477/miR-423-5p axis may be very important for the restoration of environmental regulation after SCI.

In the study of Li and colleagues, the expression of Circ 0001723 decreased significantly in rats 1 day after SCI, while the expression of miR-380-3p increased significantly. *In vitro* experiments showed that the decrease of Circ 0001723 promoted the expression of proinflammatory factors such as TNF-α and IL-1β, while the expression of miR-380-3p increased. This process inhibits the expression of HIF-1 and leads to the expression of NLRP3 and caspase-1 proteins (Li et al., 2020). Therefore, the Circ 0001723–miR-380-3p–HIF-1α axis plays a role in the regulation of inflammatory response. Circ 0001723 might be an important target related to the regulation of inflammation in SCI recovery.

The expression of these three circRNAs decreased in traumatic CNS injury (Table 2 and Figure 2). According to current research results, they all function as miRNA sponges. These three studies used different animal models. CircRNAs mentioned above are involved in different circRNA–miRNA–mRNA networks. They act on various biological processes such as inflammation, proliferation, and migration. Future research needs to confirm and explore their functions to see if they can be used clinically as therapeutic targets for the recovery from CNS post-traumatic injuries.

## Conclusion and Future Perspective

CNS post-traumatic injury is a serious public health problem. At present, there is still great value in the study of circRNAs in CNS post-traumatic injury. More and more circRNAs are being explored. Previous studies have shown that they participated in the processes of proliferation, inflammation, and apoptosis in TBI or SCI. As summarized by this review, emerging lines of evidence have described their different expressions (e.g., circRNA_16895, circRNAchr8_87,859,283–87,904,548, circRNA_01477, etc.). They act as miRNA sponges to influence the corresponding downstream mRNAs. Despite its strong clinical potential, the best way to intervene with circRNAs has not yet been defined.

Subsequent studies can be devoted to finding and confirming more functions of various circRNAs. With further research, their clinical research potential could be fully explored in the future. First, studies on the cellular level are supposed to be designed. Most of the current researches are limited to the homogenization of the whole tissue. Therefore, single-cell sequencing and other cutting-edge technologies can be applied to explore the functions of circRNAs in specific types of nerve cells or immune cells. Second, circRNAs in neuron stem cells are worth studying to find possible clinical therapeutic targets. Researches have investigated the effect of circRNA regulation in neural stem cells on functional recovery after ischemic stroke (Wang et al., 2020a). The methods could also be used in CNS post-traumatic injury. Third, the different roles of circRNAs in different recovery stages are worth exploring and comparing. Thus, we are able to find better opportunities and methods for regulation. Fourth, there is a lack of knowledge on chronic and regeneration stage of trauma. Most of the present studies have investigated the expression differences of circRNAs in the acute phase, but the chronic stage is still unknown. Fifth, circRNAs may also be used as biomarkers to monitor prognosis due to their great stability and abundance. They have been used as potential diagnostic biomarkers in other diseases such as prediabetes and tumors (Xie et al., 2017). Sixth, studies of human tissue samples have not been reported. Researches will not be confined to experimental animals in the future. The development of clinical studies is helpful to fully explore the clinical intervention value of circRNAs. Overall, future researches could maximize the benefits of circRNAs for patients suffering from CNS post-traumatic injury.

## Data Availability Statement

The original contributions presented in the study are included in the article/supplementary material, further inquiries can be directed to the corresponding author/s.

## Author Contributions

LX, XY, and L-lW wrote the draft manuscript, designed the figures, and tables, all co-authors (LX, XY, JZ, Y-yC, and L-lW) contributed substantially with revision and improvement of the manuscript. The authors read and approved the final manuscript.

## Conflict of Interest

The authors declare that the research was conducted in the absence of any commercial or financial relationships that could be construed as a potential conflict of interest.
